# Probiotics combined with prebiotics alleviated seasonal allergic rhinitis by altering the composition and metabolic function of intestinal microbiota: a prospective, randomized, double-blind, placebo-controlled clinical trial

**DOI:** 10.3389/fimmu.2024.1439830

**Published:** 2024-11-01

**Authors:** Yangfan Hou, Dan Wang, Shuru Zhou, Caifang Huo, Haijuan Chen, Fangxia Li, Minjuan Ding, Hongxin Li, Hongyan Zhao, Jin He, Hongju Da, Yu Ma, Zhihui Qiang, Xiushan Chen, Cairong Bai, Jing Cui, Na Gao, Yun Liu

**Affiliations:** ^1^ Department of Respiratory and Critical Care Medicine, Xi’an Jiaotong University Second Affiliated Hospital, Xi’an, China; ^2^ Department of Respiratory and Critical Care Medicine, Yulin No.2 Hospital, Yulin, China; ^3^ Department of Allergy, Yulin No.2 Hospital, Yulin, China

**Keywords:** allergic rhinitis, intestinal microbiota, immunity, probiotics and prebiotics, shortchain fatty acids

## Abstract

**Background:**

Numerous studies have established that probiotics or prebiotics can relieve the symptoms of allergic rhinitis (AR), but their mechanism of action remain underexplored. This study aimed to observe the clinical efficacy of probiotics combined with prebiotics in seasonal AR patients and explore their underlying mechanisms.

**Methods:**

We conducted a prospective, randomized, double-blind, placebo-controlled clinical trial. The test group was given probiotics combined with prebiotics, whereas the placebo group was administered simulated preparation for 90 days. Outcome measures included total nasal symptom score (TNSS), visual analog scale, rhinitis quality of life questionnaire, fractional exhaled nitric oxide, and the rate and intensity of Loratadine use. Serum TNF-α, INF-γ, IL-4, IL-17, and IgE levels were measured by enzyme-linked immunosorbent assay. Intestinal microbiota was detected by 16S rRNA gene sequencing and quantitative PCR. Short-chain fatty acids were analyzed by gas chromatography-mass spectrometry.

**Results:**

106 participants (N = 53 for both test group and placebo group) completed the study. From baseline to day 91, mean difference between groups (MDBG) in the reduction of TNSS was -1.1 (-2.2, -0.1) (P = 0.04); MDBG in the increment of TNF-α was 7.1 pg/ml (95% CI: 0.8, 13.4, P = 0.03); the INF-γ level was significantly increased (P = 0.01), whereas that of IL-17 (P = 0.005) was significantly decreased in the test group, whilst mean difference within groups was not statistically significant in the placebo group; MDBG in the increment of acetate was 12.4% (95% CI: 7.1%, 17.6%, P <0.001). After the administration of probiotics and prebiotics, the composition and metabolic function of the intestinal microbiota were significantly altered and positively related to the beneficial effect on seasonal AR patients.

**Conclusion:**

Probiotics combined with prebiotics administered for 90 days significantly attenuated the symptoms of seasonal AR patients, which may related to fluctuations in the composition and metabolic function of the intestinal microbiota and further ameliorating host immunity.

## Graphical Abstract

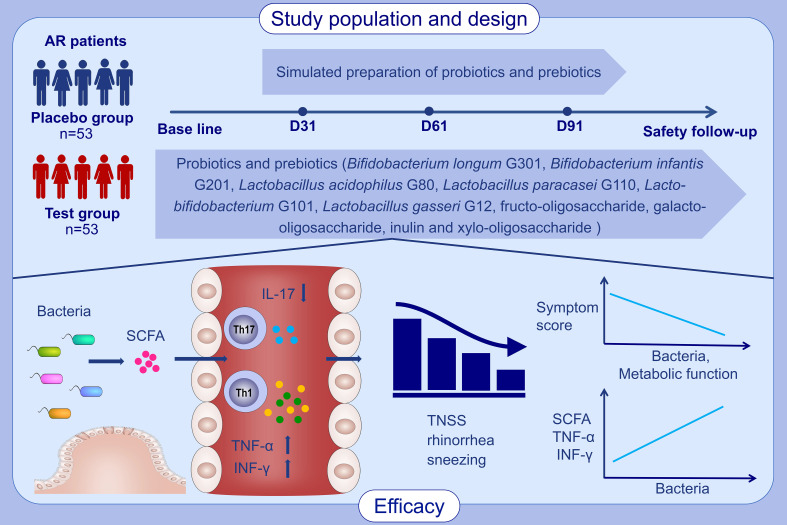

## 1 Introduction

Allergic rhinitis (AR) is one of the most frequently encountered chronic respiratory inflammatory diseases mediated by immunoglobulin E (IgE) following exposure to inhaled allergens and is characterized by sneezing, rhinorrhea, nasal pruritus, and nasal congestion. It affects an estimated 40% of the global population (1) and severely affects the quality of life, work efficiency, social life, and psychological state of patients. With its increasing incidence rate, the huge consumption of medical expenses aggravates the socioeconomic burden (2). Therefore, there is an urgent need to develop effective preventive and control strategies.

In recent years, researchers identified that the interaction between intestinal microbiota and host plays a pivotal role in the homeostasis of the immune system, and the dysbiosis of microflora can cause allergic diseases by impacting immune cells or their metabolites (3). Several studies have established that compared with healthy people, the diversity and composition of the intestinal microbiota in AR patients are significantly different, which may increase the risk of AR (4–6). Some researchers also reported that gut bacteria such as *Butyricicoccus* and *Eisenbergiella* detected in AR patients were significantly related to nasal symptoms and quality of life (7). What’s more, short-chain fatty acid (SCFA), which is produced by microbiota and potentially ameliorates the severity of allergic airway inflammation, was reduced in AR patients compared to healthy subjects (6, 8). These studies signaled that changes in the composition and function of intestinal microbiota may be linked to AR, which offers a new alternative therapeutic strategy for AR patients through microbial intervention.

At present, evidence supports the efficacy of probiotics and prebiotics in AR. For instance, Watts et al. (9) evinced that a probiotic mixture comprising 6 probiotics and 5 prebiotics improved the clinical symptoms and quality of life of AR patients and reduced the frequency of allergy-related medications by 10%. Another randomized controlled trial determined that a probiotic preparation statistically decreased the incidence of signs and symptoms of AR and limited the need for pharmacological therapy (10). However, evidence on the treatment of AR through microbial intervention is limited, and current studies are heterogeneous on the types and doses of probiotics and prebiotics and the timing of the intervention.

Immune cells participate in the occurrence of AR by secreting cytokines or immunoglobulins. When allergic reaction occurs, IL-4 secreted by type 2 T helper (Th2) cell, IL-17 secreted by type 17 T helper (Th17) cell and IgE secreted by plasma cell are increased; TNF-α and INF-γ secreted by type 1 T helper (Th1) cell are decreased (1). It is poorly understood that whether probiotic preparations can affect the pathophysiology of AR by impacting the immune system.

In order to solve the desertification in northwest China, artemisia was planted in a large area from the 1950s, which made the number of people with pollen allergy increase year by year. According to the epidemiological investigation, the content of artemisia pollen showed an upward trend from May, and with a peak from July to September (11, 12).

In this context, the clinical efficacy of probiotics combined with prebiotics on seasonal AR patients in the two northwest cities was observed via a prospective, randomized, double-blind, placebo-controlled clinical trial to explore its mechanisms from the perspective of immunity, microbial community structure, and metabolism. The primary endpoint was total nasal symptom score (TNSS) in the test and placebo groups on day 91. Secondary endpoints consisted of the scores of rhinorrhea, sneezing, nasal pruritus, and nasal congestion, the visual analog scale (VAS), rhinitis quality of life questionnaire (RQLQ), fractional exhaled nitric oxide (FeNO), and the rate and intensity of Loratadine use in the test and placebo groups on day 91. Additionally, immunological parameters (TNF-α, INF-γ, IL-17, IL-4, and IgE levels), intestinal microbiota as well as metabolite SCFA were also detected at baseline and on days 31, 61, and 91.

## 2 Materials and methods

### 2.1 Ethics approval and consent to participate

This study was approved by the Biomedical Ethics Committee of Xi’an Jiaotong University Second Affiliated Hospital and Yulin City Second Hospital (Approval no. LLGZB-XZA-004-02) and was performed in accordance with The Code of Ethics of the World Medical Association (Declaration of Helsinki). All the participants signed the informed consent form before enrolment.

### 2.2 Study design and interventions

This was a prospective, randomized, double-blind, placebo-controlled study conducted at Xi’an Jiaotong University Second Affiliated Hospital and Yulin City Second Hospital. Participants with seasonal allergic rhinitis aged 18-65 years and fulfilling the diagnostic criteria of the Chinese Society of Allergy Guidelines for Allergic Rhinitis 2018, with a course of more than 1 year, and a TNSS of more than 4, had not used antihistamines, intranasal corticosteroids, or immunosuppressants within 1 month prior to screening, were eligible to participate in the study. TNSS is a patient-assessed symptom questionnaire which consists of 4 symptoms: rhinorrhea, sneezing, nasal pruritus and nasal congestion. Each symptom was evaluated with a scale of 0, no symptoms; 1, mild; 2, moderate; 3, severe.

The exclusion criteria were as follows: history of antibiotics, antihistamines, or corticosteroids within 1 month, or probiotics and/or prebiotics in the past 6 months; asthma; tuberculosis; autoimmune disorders; severe chronic digestive diseases; and pregnant or lactating women.

The dropout criteria were as follows: the subjects stopped taking medicines or gave up participating in the trial by themselves; poor compliance of the subjects or incomplete follow-up data which affected the efficacy and safety evaluation of the medicine; subjects with major protocol violations or deviations; subjects were lost to follow-up or were pregnant; the investigators determined that patients needed to stop the study on the basis of their clinical presentation.

This experiment adopted an electronic data collection (EDC) system (DAP Software LTD, Beijing, China) to collect and manage data. According to previous study on the clinical efficacy of seasonal AR treatment (13) and the method of *Sample Size Calculations in Clinical Research (* 14), the mean of the TNSS score were expected to decrease by 1 and 2.7 respectively in the placebo group and the test group after 90 days of treatment, and the standard deviation were 2 and 3 respectively. In the SAS version 9.4 software (SAS Institutes, Cary, NC, USA), the bilateral difference test was set to α = 0.05 and β = 0.1 between the test group and placebo group, with 90% certainty, and the required sample size was 48 cases in each group. Considering a 20% attrition rate, a total of 120 patients were enrolled in this study. 120 patients with AR were planned to be enrolled and were randomly assigned to the test and placebo groups (1:1) by block randomization through the central random module of the EDC system, the block size is 4 and the blind base was stored in the system. Participants who successfully enrolled were given a random number by the research doctor and were assigned to interventions by the research nurse. Subjects, investigators, and other members of the clinical trial team were blinded during the study process from randomization to database lock-in and unblinded at the end of the study.

Patients in test group were given compound probiotics (*Bifidobacterium longum* G301, *Bifidobacterium infantis* G201, *Lactobacillus acidophilus* G80, *Lactobacillus paracasei* G110, *Lacto-bifidobacterium* G101 and *Lactobacillus gasseri* G12) (BioGrowing Co., LTD, Shanghai, China) 8g/day combined with compound prebiotics (fructo-oligosaccharide, galacto-oligosaccharide, inulin and xylo-oligosaccharide) (Quantum Hi-Tech Biological Co., LTD, Guangdong, China) 60g/day. At the same time, patients in the placebo group were given simulated preparation of probiotics (BioGrowing Co., LTD, Shanghai, China) and prebiotics (Quantum Hi-Tech Biological Co., LTD, Guangdong, China) with identical dose, packaging, label, and appearance as the test group. Treatments were consistently administered twice daily for 90 days, and patients were followed up for 30 days after the intervention. Subjects with a TNSS ≥ 8 and symptom intolerance were given Loratadine (Clarityne, Bayer Pharma Co., LTD, Shanghai, China) (10 mg once a day), and when TNSS < 8, Loratadine therapy would be discontinued. In addition, subjects were prohibited from taking imidazole antifungal agents, antibiotics, corticosteroids and immunosuppressants during this trial. Once the subject was treated with antibiotics or other drugs for irresistible reasons during the trial, it would be faithfully recorded.

The primary endpoint was TNSS in the test and placebo groups on day 91. Secondary endpoints consisted of the scores of rhinorrhea, sneezing, nasal pruritus, and nasal congestion, VAS, RQLQ, FeNO (Ruibreath, REAP Medical Technology Co., LTD, Guangzhou, China), and the rate and intensity of Loratadine use in the test and placebo groups on day 91. Additionally, serum samples were collected to detect immunological parameters (TNF-α, INF-γ, IL-17, IL-4, and IgE levels), and fecal samples were collected to analyze intestinal microbiota and metabolite SCFA at baseline and on days 31, 61, and 91. All serum samples and fecal samples were collected in sterile tubes and stored in a refrigerator at -80°C immediately after collection at different time points, and the storage time was not more than 30 days.

Subjects returned to the clinical research center for follow-up visits on days 31 (± 3 days), 61 (± 3 days), and 91 (± 3 days) after the first time taking the medications. Patients were required to return all unused medications to the study center. Investigators should promptly and accurately record the number and date of medications distributed to patients and retrieved from patients, and the actual dosage of medication used. The use of permissible medications and deviations from protocol-specified medications over the trial period were recorded by the study investigators on a case report form, and compliance was reviewed by the clinical research associate during the study visits and at the end of the study.

Adverse events and serious adverse events were monitored and recorded during the trial via physical examination, electrocardiographic examination, and laboratory testing (routine blood, urine, and fecal tests, as well as blood biochemistry).

Criteria for termination of the study were as follows: More than half of the subjects developed grade 2 or higher adverse events, or more than 1/4 of the subjects manifested grade 3 or higher adverse events; for financial, administrative, or other reasons, the sponsor required termination under the premise that the rights and safety of the subjects were fully protected; the State Medical Products Administration or the Ethics Committee ordered the suspension of the study.

### 2.3 Immunological parameters detection

Serum TNF-α, INF-γ, IL-17, IL-4, and IgE levels were measured using enzyme-linked immunosorbent assay according to the manufacturer’s protocol (Bio-Techne, Minnesota, America).

### 2.4 Intestinal microbiota profiling

Microbial DNA was extracted from fecal samples using the E.Z.N.A. DNA Kit (Omega Biotek, Norcross, GA, U.S.). Then, the 16S rDNA V3-V4 region of the eukaryotic ribosomal RNA gene was amplified by PCR using primers 341F: CCTACGGGNGGCWGCAG; 806R: GGACTACHVGGGTATCTAAT. Amplicons were extracted from 2% agarose gels and purified using an AxyPrep DNA Gel Extraction Kit (Axygen Biosciences, Union City, CA, U.S.) and quantified using QuantiFluor-ST (Promega, U.S.). Purified amplicons were then pooled in equimolar and paired-end sequenced (2 × 250) on the Illumina Hiseq2500 platform. Raw data were further filtered to acquire high-quality clean reads. Noisy sequences of raw tags were filtered by the QIIME (V1.9.1) (https://qiime2.org) pipeline to obtain high-quality clean tags. Next, the effective tags were clustered into operational taxonomic units (OTUs) of  ≥ 97% similarity using the UPARSE pipeline (http://www.drive5.com/uparse/). The representative sequences were classified into organisms by a naive Bayesian model using an RDP classifier (Version 2.2) based on the Greengenes Database (https://www.arb-silva.de/).

Quantitative PCR was performed as described in previous studies (15, 16). PCR amplification and detection were conducted with an ABI PRISM 7900-HT sequence detection system (Applied Biosystems, Foster City, CA). Standard curves were plotted based on serial dilutions of DNA extracted from known amounts of *Bifidobacterium*, *Bifidobacterium animalis*, *Lactobacillus acidophilus*, *Lactobacillus*, and *Bifidobacterium longum*. Bacterial concentrations in fecal samples were determined by comparing the Ct values acquired from standard curves.

### 2.5 Fecal SCFA profiling

Acetate, propionate, butyrate, isobutyrate, valerate, and isovalerate levels in fecal samples were analyzed via gas chromatography-mass spectrometry (GC-MS) by Zhiji Future Clinical Medical Laboratory Co., LTD. SCFAs were extracted according to the manufacturer’s protocol (BioNovoGene Technology Co., Ltd., Suzhou, China). The concentrations of SCFAs were detected using a GC-MS system (Agilent 7890/5975C, Santa Clara, CA, United States).

### 2.6 Statistical analyses

Statistical data analysis was performed using GraphPad Prism v.9 (GraphPad, California, US), SPSS 25.0 (IBM, New York, US), and R software (R Foundation, Vienna, Austria). Normally distributed measurement data were expressed as mean ± standard deviation (SD). Comparison between the two groups was made using the independent sample T-test or Wilcoxon rank sum test. The Chi-square test was used for unordered categorical variables. Covariance analysis was used to compare differences in outcome measures from baseline between the test group and placebo group.

Shannon’s alpha diversity index was calculated in QIIME. Principal coordinate analysis (PCoA) was performed and plotted in R. The functional group (guild) of the OTUs was inferred using PICRUSt (http://huttenhower.sph.harvard.edu). Differences in intestinal microbiota and function between the two groups were performed by linear discriminant analysis effect size (LEfSe). In addition, the Spearman correlation test was conducted to explore relationships between clinical indicators, intestinal microbiota, and metabolic function. P < 0.05 was considered statistically significant.

## 3 Results

### 3.1 Study population and baseline characteristics

**Figure 1:**
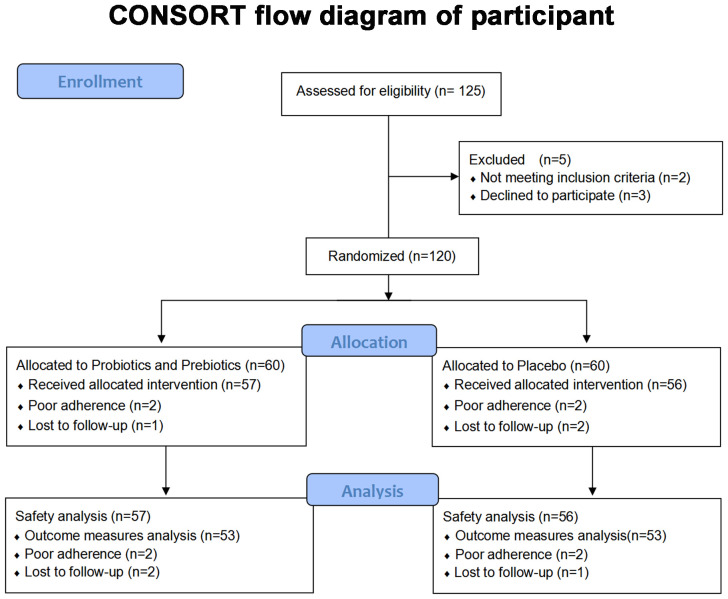
CONSORT flow diagram of participant.

125 subjects were assessed for eligibility in this trial from May 14, 2021, to November 26, 2021, and 5 were excluded given that they either did not meet the inclusion criteria or refused to participate in this trial. The flow diagram of the randomization process is illustrated in **Figure 1**. Attributable to withdrawal and poor compliance, 53 patients in both the test group and control group finally completed the trial during the pollen season. There were no serious adverse events or serious adverse reactions during the trial.

Baseline characteristics of the subjects are listed in **Table 1**. Demographics as well as the scores of TNSS, rhinorrhea, sneezing, nasal pruritus and nasal congestion, VAS, RQLQ, FeNO and IgE were similar between the test group and placebo group at baseline.

**Table 1:** Baseline characteristics of the participating subjects.

Characteristics	Probiotics and prebiotics (N=53)	Placebo (N=53)	*P*-Value
Age (years)**^†^**	37.0 ± 7.9	36.6 ± 8.3	0.68
Sex (female)**^‡^**	26 (49.1)	22 (41.5)	0.55
Height (cm)	167.6 ± 7.8	169.1 ± 7.9	0.33
Weight (Kg)	66.4 ± 11.1	67.6 ± 11.5	0.60
BMI (kg/m^2^)	23.5 ± 2.7	23.5 ± 2.9	0.97
TNSS**^†^**	7.6 ± 2.1	7.5 ± 2.6	0.68
Rhinorrhea**^†^**	2.0 ± 0.7	2.0 ± 0.9	0.92
Sneezing	1.8 ± 0.7	1.8 ± 0.8	0.90
Nasal pruritus	1.8 ± 0.8	1.9 ± 0.8	0.46
Nasal congestion**^†^**	2.0 ± 0.8	1.8 ± 0.1	0.45
VAS	20.2 ± 7.5	20.0 ± 8.6	0.89
RQLQ	81.6 ± 30.4	79.0 ± 28.6	0.65
FeNO (ppb)**^†^**	492.2 ± 239.7	520.7 ± 236.6	0.37
IgE (ng/ml)**^†^**	330.1 ± 106.4	320.1 ± 103.1	0.83

Data are expressed as mean ± SD or n (%). BMI, body mass index; TNSS, total nasal symptom score; VAS, visual analog scale; RQLQ, rhinitis quality of life questionnaire; FeNO, fractional exhaled nitric oxide; IgE, immunoglobulin E. **^†^** Wilcoxon rank-sum test; **^‡^** Chi-square test; the rest items were analyzed with independent sample t-test.

### 3.2 Changes in outcome measures and immunological parameters

The mean change in TNSS, rhinorrhea, sneezing, nasal pruritus, and nasal congestion from baseline is presented in **Table 2** and **Supplementary Figure S1**. On days 31, 61, and 91, the mean reduction in TNSS, rhinorrhea, and sneezing scores from baseline was higher in the test group than in the placebo group, and the mean difference between groups (MDBG) was statistically significant on day 91. More specifically, MDBG in the reduction of TNSS, rhinorrhea, and sneezing scores from baseline to day 91 were -1.1 (-2.2, -0.1) (*P* = 0.04), -0.3 (-0.6, -0.003) (*P* = 0.048), and -0.3 (-0.6, 0.005) (*P* = 0.054), respectively. In comparison, there was no significant difference in nasal pruritus (*P* = 0.12) and nasal congestion (*P* = 0.10) during the trial period between the two groups.

**Table 2:** Differences of TNSS and individual symptom scores between groups and within-group.

Scoring items		Baseline	D31	D61	D91
TNSS	Probiotics and prebiotics	7.6 ± 2.1	6.1 ± 2.3^***^	6.1 ± 2.9^**^	4.3 ± 3.2^***^
	Placebo	7.5 ± 2.6	6.1 ± 2.6^*^	6.3 ± 3.2	5.4 ± 2.3^***^
	Mean difference (95% confidence interval)		-0.1 (-1.0, 0.9)	-0.3 (-1.4, 0.8)	-1.1 (-2.2, -0.1)
	*P*-Value		0.89	0.61	**0.04**
Rhinorrhea	Probiotics and prebiotics	2.0 ± 0.7	1.6 ± 0.8^*^	1.5 ± 1.1^*^	1.0 ± 0.9^***^
	Placebo	2.0 ± 0.9	1.6 ± 0.9^*^	1.6 ± 0.9^*^	1.3 ± 0.7^***^
	Mean difference (95% confidence interval)		-0.03 (-0.3, 0.3)	-0.1 (-0.4, 0.2)	-0.3 (-0.6, -0.003)
	*P*-Value		0.86	0.55	**0.048**
Sneezing	Probiotics and prebiotics	1.8 ± 0.7	1.5 ± 0.7^*^	1.5 ± 0.9	1.1 ± 0.9^***^
	Placebo	1.8 ± 0.8	1.5 ± 0.7^*^	1.6 ± 0.9	1.4 ± 0.7^**^
	Mean difference (95% confidence interval)		-0.06 (-0.3, 0.2)	-0.03 (-0.4, 0.3)	-0.3 (-0.6, 0.005)
	*P*-Value		0.68	0.88	**0.054**
Nasal pruritus	Probiotics and prebiotics	1.8 ± 0.8	1.5 ± 0.7	1.5 ± 0.8	1.0 ± 1.0^***^
	Placebo	1.9 ± 0.8	1.5 ± 0.8^*^	1.6 ± 1.0	1.4 ± 0.8^**^
	Mean difference (95% confidence interval)		0.05 (-0.2, 0.3)	0.007 (-0.3, 0.3)	-0.3 (-0.6, 0.07)
	*P*-Value		0.73	0.97	0.12
Nasal congestion	Probiotics and prebiotics	1.9 ± 0.8	1.5 ± 0.8^**^	1.55 ± 0.911^*^	1.2 ± 0.9^***^
	Placebo	1.8 ± 0.1	1.5 ± 1.0	1.7 ± 1.0	1.4 ± 0.9^*^
	Mean difference (95% confidence interval)		-0.04 (-0.4, 0.3)	-0.2 (-0.5, 0.2)	-0.3 (-0.6, 0.06)
	*P*-Value		0.82	0.31	0.10

Data are expressed as mean ± SD. Wilcoxon rank sum test or t-test was used to compare differences in symptom scores from baseline within groups; covariance analysis was used to compare differences in symptom scores from baseline between two groups. ^*^ *P* < 0.05, ^**^ *P* < 0.01, ^***^ *P* < 0.001 for the difference from baseline within groups. TNSS, total nasal symptom score.

The bold values mean statistical significance.

MDBG in the change from baseline in the scores of VAS and RQLQ, FeNO level, and the rate and intensity of Loratadine use was not significant during this trial. However, the scores of VAS and RQLQ in the test group had a greater tendency to decline compared to the placebo group on day 91. (see **Supplementary Tables S1, S2**).

Changes in immunological parameters from baseline in the test group and placebo group are summarized in **Table 3**. For instance, MDBG in the increment of TNF-α was 7.1 pg/ml (95% CI: 0.8, 13.4, P = 0.03) on day 91 from baseline; mean difference within groups (MDWG) in the increment of TNF-α was 13.9 ± 13.9 pg/ml (95% CI: 10.1, 17.8, P = 0.002) in the test group on day 61 from baseline. In addition, the INF-γ level was significantly increased (*P* = 0.01), whereas that of IL-17 (*P* = 0.005) was significantly decreased on day 91 compared to the baseline level in the test group, whilst MDWG at different time points from baseline was not statistically significant in the placebo group.

**Table 3:** Differences of immunological parameters between groups and within-group.

Items		Baseline	D31	D61	D91
TNF-α (pg/ml)	Probiotics and prebiotics	86.6 ± 20.1	87.0 ± 20.2	100.5 ± 20.5^**^	87.6 ± 21.0
	Placebo	88.3 ± 21.3	84.8 ± 26.9	98.5 ± 27.8	81.8 ± 25.3
	Mean difference (95% confidence interval)		3.4 (-3.0, 9.8)	2.8 (-4.6, 10.2)	7.1 (0.8, 13.4)
	*P*-Value		0.29	0.46	**0.03**
INF-γ (pg/ml)	Probiotics and prebiotics	503.8 ± 200.5	670.2 ± 238.4^***^	473.4 ± 202.9	595.3 ± 157.8^*^
	Placebo	489.3 ± 211.8	637.7 ± 266.5	466.5 ± 206.2	570.3 ± 199.3
	Mean difference (95% confidence interval)		21.0 (-59.6, 101.6)	-2.0 (-71.5, 67.6)	18.159 (-40.5, 76.8)
	*P*-Value		0.61	0.95	0.54
IL-17 (pg/ml)	Probiotics and prebiotics	335.7 ± 82.3	313.6 ± 73.4	321.4 ± 67.0	292.5 ± 65.1^**^
	Placebo	337.1 ± 80.2	315.9 ± 85.8	311.9 ± 89.3	275.5 ± 78.9
	Mean difference (95% confidence interval)		-2.5 (-24.8, 19.8)	9.1 (-15.6, 33.7)	17.9 (-1.3, 37.1)
	*P*-Value		0.83	0.47	0.07
IL-4 (pg/ml)	Probiotics and prebiotics	38.3 ± 11.1	49.5 ± 13.0^***^	40.7 ± 9.9	42.3 ± 8.6
	Placebo	37.2 ± 11.6	48.2 ± 14.0	40.6 ± 12.0	41.2 ± 11.6
	Mean difference (95% confidence interval)		0.4 (-3.6, 4.5)	-0.6 (-4.1, 2.9)	0.6 (-2.4, 3.6)
	*P*-Value		0.84	0.74	0.71
IgE (ng/ml)	Probiotics and prebiotics	330.1 ± 106.4	344.6 ± 90.3	334.2 ± 91.3	337.6 ± 85.1
	Placebo	320.1 ± 103.1	324.4 ± 108.8	331.2 ± 114.6	325.6 ± 118.5
	Mean difference (95% confidence interval)		11.5 (-11.1, 34.2)	-4.8 (-35.1, 25.5)	4.3 (-20.9, 29.6)
	*P*-Value		0.32	0.75	0.73

Data are expressed as mean ± SD. The Wilcoxon rank sum test or t-test was used to compare differences between groups at baseline and within groups; covariance analysis was used to compare differences in immunological parameters from baseline between groups. ^*^ *P* < 0.05, ^**^ *P* < 0.01, ^***^ *P* < 0.001 for the difference from baseline within groups. TNF-α, tumor necrosis factor-α; INF-γ, interferon-gamma; IL-17, interleukin 17; IL-4, interleukin 4; IgE, immunoglobulin E.

The bold values mean statistical significance.

### 3.3 MDBG in intestinal microbiota at different time points

**Figure 2:**
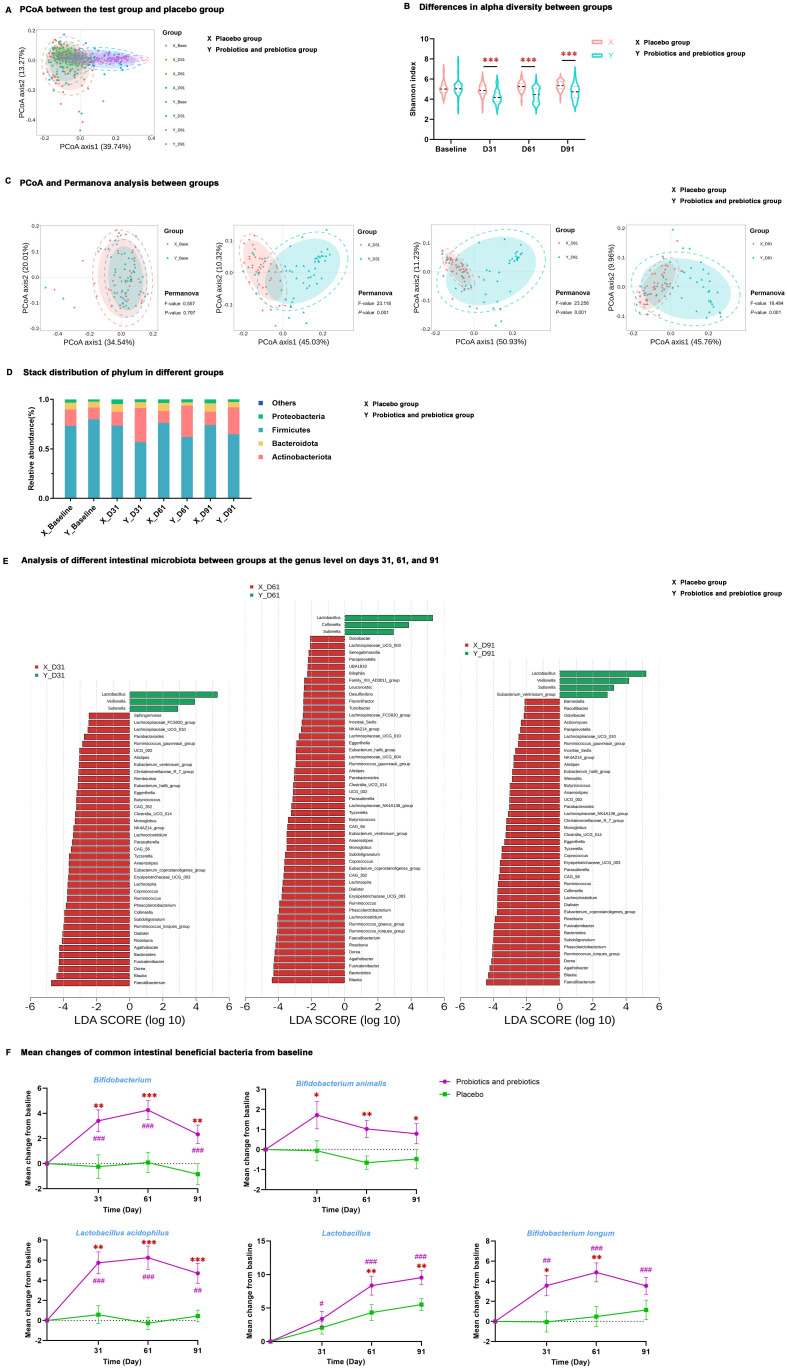
Differences in intestinal microbiota at different time points between the test group and placebo group. **(A)** PCoA between the test group and placebo group. **(B)** Differences in alpha diversity between groups. **(C)** PCoA and Permanova analysis between groups. **(D)** Stack distribution of phylum in different groups. **(E)** Analysis of different intestinal microbiota between groups at the genus level on days 31, 61, and 91. **(F)** Mean changes of *Bifidobacterium*, *Bifidobacterium animalis*, *Lactobacillus acidophilus*, *Lactobacillus*, and *Bifidobacterium longum* from baseline; data are expressed as mean ± SE. ^#^ *P* < 0.05, ^##^ *P* < 0.01, ^###^ *P* < 0.001 for the difference from baseline within groups. ^*^ *P* < 0.05, ^**^ *P* < 0.01, ^***^ *P* < 0.001 for the difference between groups. PCoA, principal coordinate analysis; X, placebo group; Y, probiotics and prebiotics group.

16S rRNA gene sequencing revealed that intestinal microbiota from samples of the test group at baseline and all samples of the placebo group displayed an aggregation distribution, while the intestinal microbiota from the test group on days 31, 61, and 91 presented another aggregation distribution (**Figure 2A**). MDBG in alpha (α) diversity (determined by the Shannon index) and beta (β) diversity (determined by Principal coordinate analysis and Permanova analysis) was not significant at baseline, but α diversity was significantly decreased while β diversity was significantly increased in the test group compared to placebo group during the trial period (*P* < 0.001) (**Figures 2B, C**).

MDBG in microbiota abundance was examined at the phylum and genus levels, and there was no significant difference at baseline. However, at the phylum level, the test group had a lower proportion of *Firmicutes* and a higher proportion of *Actinobacteria* than the placebo group on days 31, 61, and 91 (*P* < 0.01) (**Figure 2D**, **Supplementary Table S3**). LEfSe at the genus level determined that after probiotics and prebiotics treatment, *Lactobacillus*, *Sutterella*, *Veillonella*, and *[Eubacterium]_ventriosum_group* became the dominant microbiome in the test group, whereas *Faecalibacterium*, *Bacteroides*, *Blautia*, *Agathobacter*, *Dorea*, [*Ruminococcus*]_*torques*_*group* and *Subdoligranulum* were the dominant microbiome in the placebo group (*P* < 0.05) (**Figure 2E**).

Quantification changes were detected in five common intestinal beneficial bacteria from baseline by qPCR (**Figure 2F**, **Supplementary Table S4**), and there was no significant difference between the test and placebo groups at baseline. MDWG in these five beneficial bacteria on days 31, 61, and 91 from baseline was not significant in the placebo group. MDBG in the increment of *Bifidobacterium, Bifidobacterium animalis*, and *Lactobacillus acidophilus* on days 31, 61, and 91 from baseline was remarkable (*P* < 0.05). Lastly, there was a significant increase in *Lactobacillus* on days 61 and 91 and *Bifidobacterium longum* on days 31 and 61 from baseline in the test group compared to the placebo group (*P* < 0.05).

### 3.4 Function prediction of microbiota and changes in metabolites SCFA

**Figure 3:**
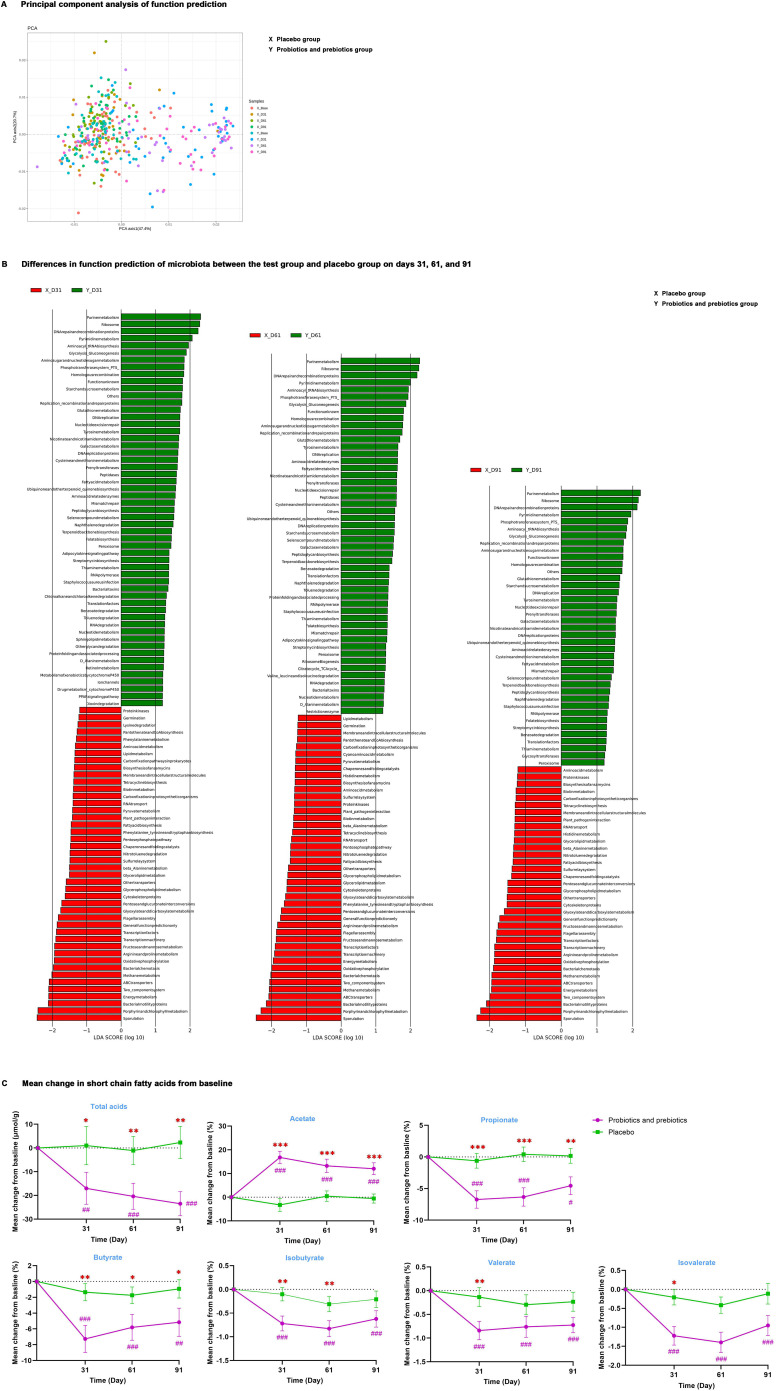
The function of intestinal microbiota. **(A)** Principal component analysis of function prediction. **(B)** Differences in function prediction of microbiota between the test group and placebo group on days 31, 61, and 91. **(C)** Mean change in total acids and percentages of acetate, propionate, butyrate, isobutyrate, valerate, and isovalerate from baseline; data are expressed as mean ± SE. Covariance analysis was used to compare differences from baseline levels between the two groups. ^#^ *P* < 0.05, ^##^ *P* < 0.01, ^###^ *P* < 0.001 for the difference from baseline within groups. ^*^ *P* < 0.05, ^**^ *P* < 0.01, ^***^ *P* < 0.001 for the difference between groups. X, placebo group; Y, probiotics and prebiotics group.

PICRUSt analysis was carried out to predict the Kyoto Encyclopedia of Genes and Genomes (KEGG) functional pathways of intestinal microbiota. Principal component analysis (**Figure 3A**) determined that microbiota function in the test group was similar to that of the placebo group at baseline; after probiotics and prebiotics treatment, MDBG in microbiota function analyzed by LEfSe (**Figure 3B**) on days 31, 61 and 91 was significant. At the study endpoint, 41 differential metabolic pathways were identified between the two groups. Specifically, enriched pathways in the test group chiefly included purine metabolism, pyrimidine metabolism, glycolysis/gluconeogenesis, amino sugar and nucleotide sugar metabolism, starch and sucrose metabolism, and tyrosine metabolism.

Then, common microbial metabolite SCFAs were detected (**Figure 3C**, **Supplementary Table S5**), and MDBG was not significant at baseline and MDWG of SCFAs on days 31, 61, and 91 compared to baseline was also not remarkable in the placebo group. After probiotics and prebiotics treatment, the percentage of acetate was remarkably increased by 12.4% (95% CI: 7.1%, 17.6%, *P <*0.001), while the concentration of total acids and the proportion of propionate and butyrate were significantly decreased (*P* < 0.05) from baseline in the test group compared with the placebo group on days 31, 61 and 91. In addition, MDBG in the decreases of the percentage of isobutyrate on days 31 and 61 and of the percentage of valerate and isovalerate on day 31 compared to baseline was noted (*P* < 0.05), despite their percentage in SCFAs not being high.

### 3.5 Correlations between clinical indicators, intestinal microbiota, and metabolic function

**Figure 4:**
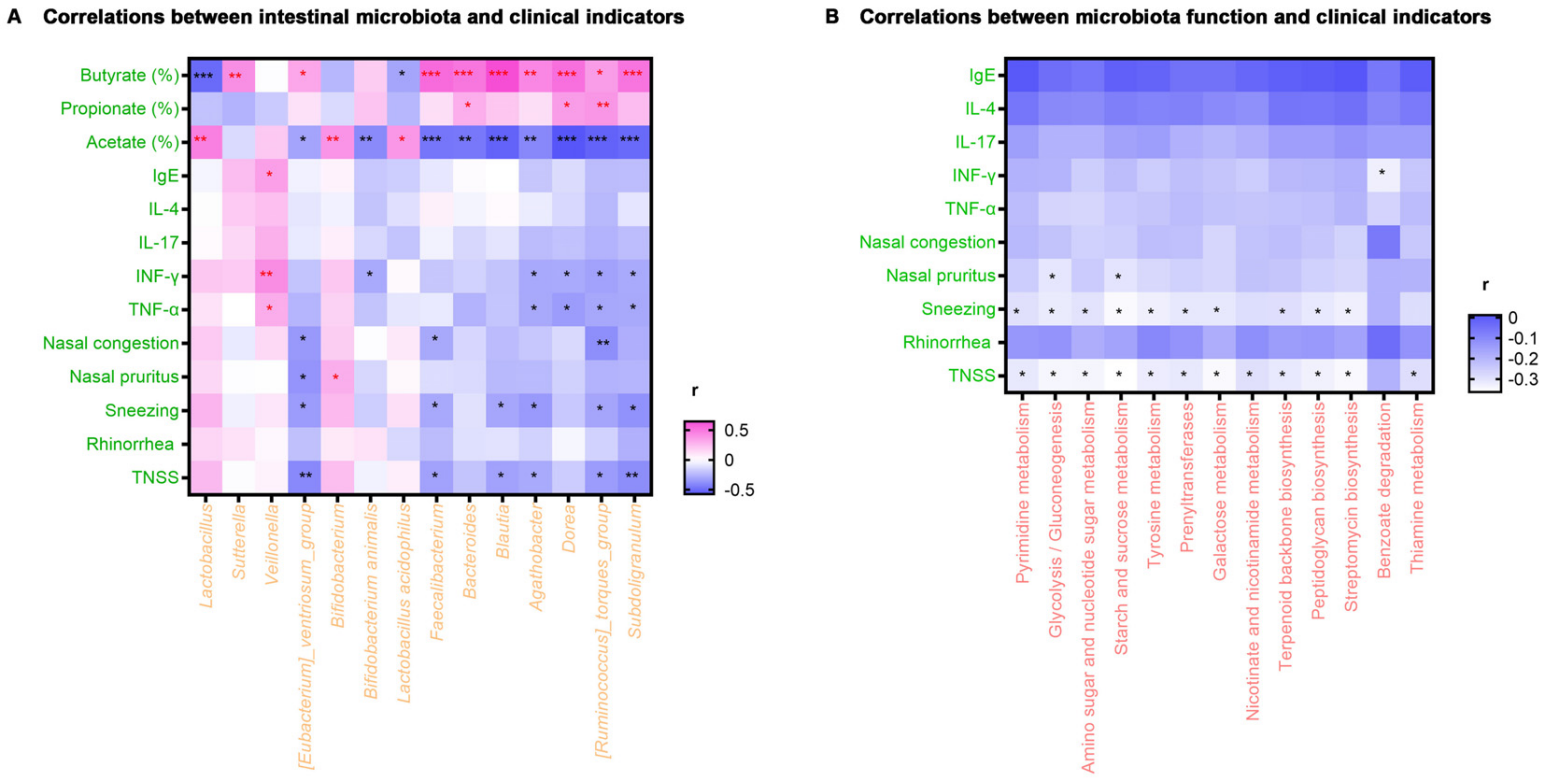
Heat map of correlation analysis. **(A)** Correlations between intestinal microbiota and clinical indicators. **(B)** Correlations between microbiota function and clinical indicators. ^*^ *P* < 0.05, ^**^ *P* < 0.01, ^***^ *P* < 0.001. r, related coefficient; TNSS, total nasal symptom score. Red asterisks represent positive correlations, and black asterisks represent negative correlations.

Spearman rank correlation analysis was employed to investigate the correlations between alterations in clinical characteristics of AR patients at the study endpoint and fluctuations in intestinal microbiota induced by probiotics and prebiotics treatment. As displayed in **Figure 4A**, scores of TNSS and sneezing were negatively correlated with *[Eubacterium]_ventriosum_group, Faecalibacterium*, *Blautia*, *Agathobacter*, *[Ruminococcus]*_*torques*_*group* and *Subdoligranulum* (*P* < 0.05). Meanwhile, the nasal pruritus score was negatively correlated with *[Eubacterium]_ventriosum_group* (*P* < 0.05), while the nasal congestion score was negatively correlated with *[Eubacterium]_ventriosum_group*, *Faecalibacterium*, and *[Ruminococcus]*_*torques*_*group* (*P* < 0.05). Besides, the level of TNF-α and IFN-γ were positively correlated with *Veillonella* and negatively correlated with *Agathobacter*, *Dorea*, *[Ruminococcus]*_*torques*_*group*, and *Subdoligranulum* (*P* < 0.05). Regarding SCFA, the percentage of acetate was positively correlated with *Lactobacillus, Bifidobacterium*, and *Lactobacillus acidophilus*, while the percentage of propionate was positively correlated with *Bacteroides*, *Dorea*, and *[Ruminococcus]*_*torques*_*group* (*P* < 0.05). Additionally, *[Eubacterium]_ventriosum_group*, *Faecalibacterium*, *Bacteroides*, *Blautia*, *Agathobacter*, *Dorea*, *[Ruminococcus]*_*torques*_*group*, and *Subdoligranulum* were positively correlated with the percentage of butyrate (*P* < 0.05).

Relationships between the clinical indicators and microbiota metabolic function enriched in the test group were also investigated. As exhibited in **Figure 4B**, scores of TNSS and sneezing were negatively correlated with pyrimidine metabolism, glycolysis/gluconeogenesis, amino sugar and nucleotide sugar metabolism, starch and sucrose metabolism, tyrosine metabolism, prenyl transferases, galactose metabolism, terpenoid backbone biosynthesis, peptidoglycan biosynthesis and streptomycin biosynthesis (*P* < 0.05). On the other hand, the TNSS score was negatively correlated with the metabolism of nicotinate, nicotinamide, and thiamine (*P* < 0.05). Finally, the nasal pruritus score was negatively correlated with glycolysis/gluconeogenesis and starch and sucrose metabolism (*P* < 0.05).

## 4 Discussion

The microbiome plays a critical role in the development and maturation of the host’s innate and adaptive immune system (17). Consequently, disturbances in intestinal microbiota may cause airway allergic diseases by altering the host immune system, and recently, the modulation of intestinal microbiota has been considered a potential alternative or adjuvant therapy for airway allergic diseases (18). Indeed, a multitude of clinical trials have established that probiotics or prebiotics can relieve the symptoms of allergic rhinitis (19–21), but studies exploring the underlying mechanism of action are limited. This study observed the effect of probiotics combined with prebiotics in adult seasonal AR patients and further examined their possible mechanism by dynamically monitoring alterations in clinical symptoms, immunological indicators, and the composition and metabolic function of the intestinal microbiota of subjects.

Compared with the placebo group, probiotics combined with prebiotics administered for 90 days significantly attenuated the symptoms of AR patients in the present study. TNSS and the scores of rhinorrhea and sneezing were reduced, while no statistically significant differences were observed in the scores of VAS and RQLQ, FeNO level, and the rate and intensity of Loratadine use. We noticed that the scores of clinical symptoms of AR patients were steady declined during the trial in both the test group and placebo group, which may be a consequence of the natural pathophysiological progression of AR because the patient’s condition changed dynamically over time. The clinical symptoms of AR patients in the test group were relieved more significant than those in the placebo group, therefore supplementation with probiotics and prebiotics accelerated the improvement of AR patients. In addition, the slowing relief degree of clinical symptoms on day 61 may be associated with the increase of pollen concentration. Similarly, because autumn is the peak of exposure to artemisia pollen, the intensity of Loratadine use was increased in both the test group and placebo group on day 61.

Our results were consistent with those of previous studies that found that probiotic supplements can alleviate AR and improve patients’ clinical symptoms. For instance, *Lactobacillus helveticus* SBT2171 limited the severity of perennial AR in adults and suppressed eosinophil counts in both the blood and nasal fluids (21). Similarly, a *Bifidobacterium* mixture (B longum BB536, B infantis M-63, and B breve M-16V) significantly improved AR symptoms and quality of life in children with pollen-induced AR (22). Besides, a probiotic supplement containing *Bifidobacterium animalis* Subsp. *Lactis* BB12, *Enterococcus faecium* L3, oligofructose, and fatty acids relieved nasal symptoms and minimized the intake of oral antihistamines and inhaled corticosteroids (10).

AR is caused by a disruption in the Th1/Th2 ratio. Activated allergen-specific Th2 cells can activate the inflammatory cascade by secreting type 2 cytokines such as IL-4, IL-5, and IL-13, thereby exacerbating allergic symptoms (2). In addition, Th17 cells are involved in the pathophysiology of AR by secreting IL-17, which impacts the severity of the disease (23). According to the observations of multiple clinical and animal studies, bacterial lysates such as OM85-Broncho-Vaxom, and probiotics such as *Lacticaseibacillus paracasei* GM-080 and *Lactiplantibacillus plantarum* NR16 can relieve AR symptoms by increasing the levels of Th1 cytokines and/or decreasing the levels of Th2 cytokines (24–26). In this study, probiotics combined with prebiotics increased TNF-α and INF-γ levels and decreased IL-17 levels on day 91. Considering that INF-γ is a potent inhibitor of Th17 cells (27), this intervention may be conducive to the secretion of Th1 cytokines (TNF-α and INF-γ) to modulate the Th1/Th2 balance and lower the levels of Th17 cytokines (IL-17) to alleviate AR symptoms.

What’s more, the increase in IL-4 from the baseline in both groups on day 31 may be a manifestation of AR progression because of the increase of pollen exposure. On day 31, TNF-α and INF-γ in the test group were higher than that in the baseline period, and therefore the increase of IL-4 in the test group may be the body’s natural feedback to maintain the balance of Th1/Th2. However, MDBG in the increment of IL-4 was only 0.4, and this result was not statistically significant. Therefore, the combination of probiotics and prebiotics did not significantly affect the expression of IL-4 in this trial.

However, our results were not in agreement with some previous studies. For example, Mårtensson A et al. (28) described that administration of a probiotic assemblage comprising *Lactobacillus rhamnosus* SP1, *Lactobacillus paracasei* 101/37, and *Lactococcus lactis* L1A exerted minimal effects in patients with seasonal AR in terms of quality of life and signs and symptoms of AR. In a four-week clinical trial, a mixture of *Bifidobacterium longum* and *Lactobacillus plantarum* decreased IgE levels and increased IL-10 levels in perennial AR subjects, but no significant change in the levels of IL-4, IL-5, IL-13, and INF-γ was noted (29). Disparities in these results may be attributed to the contrasting study designs, such as the allergen, population demographics, severity of symptoms, the types and doses of probiotics or prebiotics, and duration of intervention.

In order to explore the impact of probiotics and prebiotics in the development of AR by altering the gut flora, fecal microorganisms of the study population were investigated. The results exposed that the intestinal microbiota communities in the test group on days 31, 61, and 91 were similar, and all of them were significantly different from that of baseline, while no significant difference was observed in the placebo group, implying that the intervention played an effective role on intestinal microbiota. After the intervention, α diversity was significantly decreased, whereas β diversity was significantly increased in AR patients. In an observational study on gut bacteria, Zhu L et al. (26) discovered that healthy controls possessed lower α diversity and higher β diversity in comparison with AR patients; they (30) also found that the symptoms were relieved and α diversity was decreased in AR patients after a herbal formula treatment, which is in line with the results of this study in the sense that intervention may restore intestinal microbiota diversity in AR patients. However, the association between microbial diversity and AR remains controversial. Some researchers evinced that AR patients had lower or similar microbiota diversity compared with healthy individuals (4, 5, 31). This may be related to the heterogeneity of studies, given that the intestinal flora is affected by various factors such as genetics, age, gender, and dietary habits of hosts from different regions.

In terms of microbiota composition, the abundance of *Firmicutes* was reduced, and that of *Actinobacteria* was increased at the phylum level following intervention with probiotics combined with prebiotics. Meanwhile, at the genus level, the abundance of beneficial bacteria, including *Lactobacillus*, *Sutterella*, *Veillonella, [Eubacterium]_ventriosum_group, and Bifidobacterium* were significantly enriched, whereas that of *Faecalibacterium*, *Bacteroides*, *Blautia*, *Agathobacter*, *Dorea*, *[Ruminococcus]*_*torques*_*group* and *Subdoligranulum* were significantly decreased.

Earlier studies compared gut microbiota between AR patients and healthy individuals and determined that in AR group, *Actinobacteria* was decreased; *Firmicutes* and *Bacteroidete* were enriched, which may promote allergen sensitization and were associated with AR development; *Lactobacillus, Sutterella*, *Veillonella*, and *Bifidobacterium* was decreased, whereas that of *Faecalibacterium*, *Roseburia*, and *Blautia* was increased at the genus level (4, 5, 7, 32, 33). Consistent with the observations of the current study, prior studies also found that the abundance of *Actinobacteria* and *Lactobacillus* was increased, and that of *Bacteroides* was reduced in AR patients treated with probiotic preparations (34, 35). Furthermore, the microbiota functional pathway of the test group was significantly different from that of the placebo group, and purine metabolism, pyrimidine metabolism, glycolysis/gluconeogenesis, amino sugar and nucleotide sugar metabolism, starch and sucrose metabolism, tyrosine metabolism, and peptidoglycan biosynthesis were the significantly enriched pathways. Purine metabolism and peptidoglycan biosynthesis were also validated to be more prevalent among healthy controls compared with AR patients (5). Therefore, our study demonstrated that probiotics combined with prebiotics could restore intestinal microbiota and its metabolic function in AR patients.

In order to further explore the influence of microbiota and its function in AR and the underlying mechanism, the correlations between clinical indicators, probiotics, and microbial metabolic function were analyzed. The results demonstrated that bacteria including *[Eubacterium]_ventriosum_group, Faecalibacterium, Blautia, Agathobacter, [Ruminococcus]_torques_group and Subdoligranulum* and major metabolic pathways were negatively correlated with scores of TNSS, sneezing, nasal pruritus, and nasal congestion, insinuating that probiotics combined with prebiotics can alleviate AR symptoms by adjusting intestinal microbiota and its metabolic function. Bacterial metabolites can affect immune cells or penetrate the lung through the circulatory system and affect pulmonary inflammation through the gut-lung axis (36). Increased percentage of acetate and enriched purine metabolism (inosine), nicotinamide metabolism and tyrosine metabolism in our test group have been found to exert a protective effect against allergic airway inflammation in previous studies (37–39). Herein, the enriched *Lactobacillus*, *Bifidobacterium*, and *Lactobacillus acidophilus* were positively correlated with the percentage of acetate; likewise, the enriched *Sutterella* and *[Eubacterium]*_*ventriosum*_group was positively correlated with the percentage of butyrate. As is well documented, acetate and butyrate are the most abundant SCFAs produced by intestinal flora fermenting dietary fiber and are hypothesized to exert a protective effect against allergic airway inflammation by modulating the activity of T cells and dendritic cells and decreasing levels of circulating IgE (37, 40). In this trial, probiotics combined with prebiotics increased the percentage of acetate and decreased the percentage of butyrate in the test group, which may related to the specific species of probiotics we supplemented. The probiotics supplemented in this trial belong to *Bifidobacterium* and *Lactobacillus*, which mainly produce acetate. Therefore, when the acetate-producing bacteria significantly enriched, the butyrate-producing bacteria may became the non-dominant bacteria in the gut. These results remind us that supplementation with both acetate-producing probiotics and butyrate-producing probiotics may provide greater benefit to AR patients.

As for the immunity indicator, on the one hand, enriched *Veillonella* was positively correlated with TNF-α and INF-γ levels, signifying that *Veillonella* may promote the function of Th1 cells. As a key component of gut bacterium, *Veillonella* may exert a protective role in immune system development and is negatively correlated with asthma (41, 42). On the other hand, bacteria *Agathobacter*, *Dorea*, *[Ruminococcus]*_*torques*_*group*, and *Subdoligranulum* negatively correlated with the percentage of acetate were also negatively correlated with the levels of TNF-α, and INF-γ. Previous studies demonstrated that acetate can activate Th1 cells by inhibiting histone deacetylase (43, 44). Therefore an increase in the percentage of acetate in the test group may stimulate the secretion of TNF-α and INF-γ. To sum up, changes in microbiota and its metabolic function may alleviate AR by affecting host immunity.

It is worth noting that the composition of intestinal microbiota, the functional pathway of bacteria, and metabolite SCFA were significantly altered since the 31st day, whereas alterations in immunological indicators and significant relief of AR symptoms were observed on the 91st day, inferring that probiotics combined with prebiotics may regulate intestinal microbiota and its metabolites and subsequently changing the host immune state to alleviate AR. Owing to the complexity of the intestinal microecosystem, the pathway through which specific bacteria and their metabolites affect the immune system remains to be further explored in the future.

There are some limitations that cannot be overlooked in the present study. To begin, since intestinal microbiota is affected by the living environment and dietary habits of the host, the inclusion of healthy controls would allow the comparison of the intestinal microbiota of AR patients to that of healthy individuals after receiving probiotic preparations. Secondly, fecal samples provide information on non-absorbed SCFAs, and SCFAs in plasma may better reflect their role in the respiratory tract (45). Thirdly, significant changes only in the levels of Th1 and Th17 cytokines were observed at the end of this study, and extending treatment time with probiotics and prebiotics may be an effective strategy to discover more significant changes in immune indicators.

To the best of our knowledge, this is the first prospective cohort study to dynamically monitor clinical indicators, immune indicators, intestinal microbiota and its functional pathways, and bacterial metabolite SCFAs in seasonal AR patients treated with probiotics combined with prebiotics. The results showed that probiotics combined with prebiotics significantly relieved the symptoms of AR patients, increased the level of Th1 cytokines, changed the composition and metabolic function of the intestinal microbiota, and significantly increased the percentage of acetate. Furthermore, correlation analysis revealed that this beneficial effect might result from changing the composition and metabolic function of intestinal microbiota and further changing host immunity via the gut-lung axis. This provides a theoretical basis for the future application of compound probiotic and prebiotic preparations as an alternative therapy for seasonal AR patients.

## Data Availability

The datasets presented in this study can be found in online repositories. The names of the repository/repositories and accession number(s) can be found in the article/**Supplementary Material.**
