# Supplementary material for: Probiotics combined with prebiotics alleviated seasonal allergic rhinitis by altering the composition and metabolic function of intestinal microbiota: a prospective, randomized, double-blind, placebo-controlled clinical trial
Source: Front Immunol. 2024 Nov 1;15:1439830. doi: 10.3389/fimmu.2024.1439830 (PMC11563974; doi:10.3389/fimmu.2024.1439830)
Supplement: Supplementary file 1 [file DataSheet1.pdf]

Supplementary Material

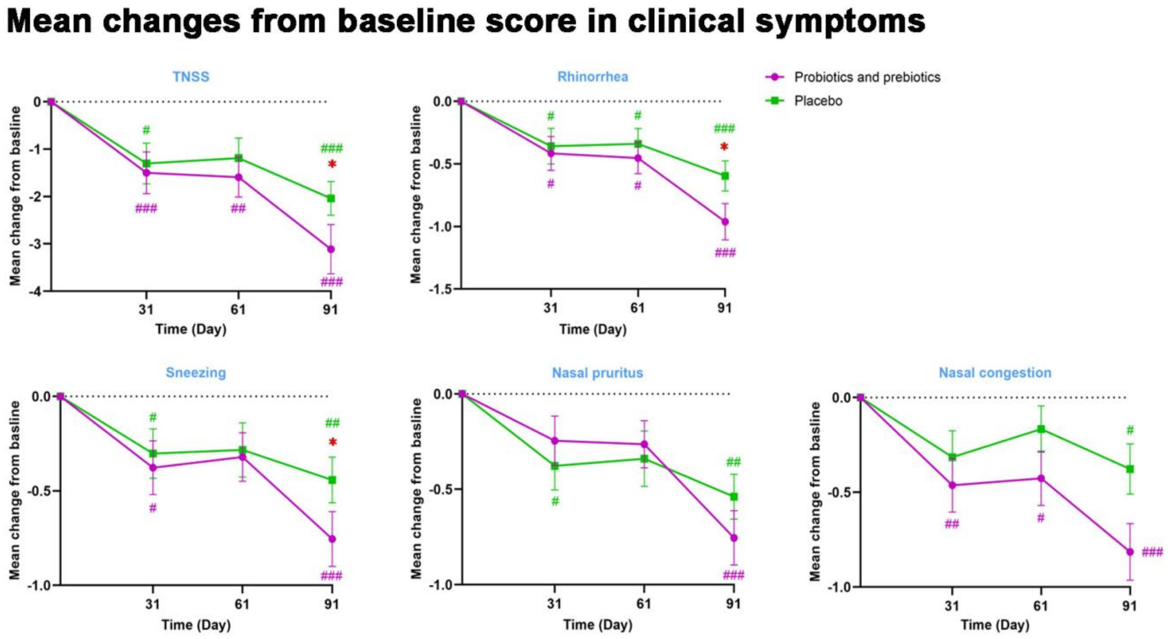

Fig. S1 Mean change from baseline score in clinical symptoms on days 31, 61, and 91. **A,** Mean change of TNSS. **B,** Mean change of rhinorrhea. **C,** Mean change of sneezing. **D,** Mean change of nasal pruritus. **E,** Mean change of nasal congestion. Data are expressed as mean ± SE. Covariance analysis was used to compare differences from baseline scores between the two groups. **#** $P < 0.05$, **##** $P < 0.01$, **###** $P < 0.001$ for the difference from baseline within groups. ***** $P < 0.05$ for the difference between groups. TNSS, total nasal symptom score.

Table S1 Differences of VAS, RQLQ and FeNO between groups and within-group

Items		Baseline	D31	D61	D91
VAS	Probiotics and prebiotics	20.2±7.5	15.8±7.9**	15.3±9.1**	10.8±9.7***
	Placebo	20.0±8.6	15.1±8.4**	16.4±9.7*	13.1±7.2***
	Mean difference		0.6	-1.1	-2.3
	(95% confidence interval)		(-2.5, 3.7)	(-4.7, 2.4)	(-5.5, 0.8)
	P-Value		0.69	0.53	0.14
RQLQ	Probiotics and prebiotics	81.6±30.4	65.5±28.1**	73.7±37.4	50.8±40.3***
	Placebo	79.0±28.6	66.0±37.9	69.1±42.3	52.4±31.2***
	Mean difference		-1.9	3.0	-2.7
	(95% confidence interval)		(-13.3, 9.6)	(-10.9, 17.0)	(-15.7, 10.2)
	P-Value		0.75	0.67	0.68
FeNO (ppb)	Probiotics and prebiotics	492.2±239.7	461.0±252.3	454.6±183.2	393.1±152.7*
	Placebo	520.7±236.6	496.3±229.1	456.6±181.8	389.3±174.0**
	Mean difference		-24.1	19.0	10.7
	(95% confidence interval)		(-112.2, 63.9)	(-46.5, 84.4)	(-49.7, 71.2)
	P-Value		0.59	0.57	0.73

Data are expressed as mean ± SD. Wilcoxon rank sum test or t-test was used to compare differences in scores of VAS and RQLQ, and FeNO level from baseline within groups; covariance analysis was used to compare differences on days 31, 61, and 91 from baseline between the two groups. * $P < 0.05$, ** $P < 0.01$, *** $P < 0.001$ for the difference from baseline within groups. VAS, visual analog scale; RQLQ, rhinitis quality of life questionnaire; FeNO, fractional exhaled nitric oxide.

Table S2 Rate and intensity of Loratadine use between groups during the trial period

Items		0-30 days	31-60 days	61-90 days	0-90 days
Rate of Loratadine use† (%)	Probiotics and Prebiotics	35.2	37.0	44.4	64.8
	Placebo	50.0	46.2	34.6	63.5
	P-Value	0.12	0.34	0.30	0.88
Intensity of Loratadine use‡	Probiotics and prebiotics	0.1±0.2	0.3±0.6	0.2±0.5	0.2±0.4
	Placebo	0.3±0.4	0.4±0.6	0.2±0.4	0.3±0.4
	P-Value	0.08	0.31	0.44	0.66

Data are % or mean ± SD. †, Chi-square Test; ‡, Wilcoxon rank-sum test.

Table S3 Differences in microbiota abundance between groups at phylum level

Species name	Time point	Baseline	D31	D61	D91
Firmicutes (%)	Probiotics and prebiotics	78.7	56.7	62.3	64.5
	Placebo	72.9	73.1	76.1	73.9
	P-Value	0.06	**0.001**	**0.001**	**0.007**
Actinobacteria (%)	Probiotics and prebiotics	12.7	34.8	31.1	27.3
	Placebo	16.5	14.1	12.1	13.5
	P-Value	0.18	**0.001**	**0.001**	**0.001**

Data are expressed as mean abundance. Metastats analysis was used to compare differences between groups at different time points.

Table S4 Differences of intestinal beneficial bacteria between groups and within-group

Bacteria		Baseline	D31	D61	D91
Bifidobacterium	Probiotics and prebiotics	9.8±5.6	13.2±4.2***	14.0±2.9***	12.1±4.8***
	Placebo	10.9±4.7	10.5±5.3	10.9±4.1	10.0±5.2
	Mean difference		3.5	4.1	3.1
	(95% confidence interval)		(1.1, 5.9)	(2.0, 6.1)	(0.9, 5.2)
	P-Value		**0.004**	**<0.001**	**0.005**
Bifidobacterium animalis	Probiotics and prebiotics	0.3±2.0	2.0±4.5	1.3±3.4	1.1±2.9
	Placebo	0.8±3.0	0.6±2.3	0.2±1.3	0.3±1.8
	Mean difference		1.8	1.7	1.3
	(95% confidence interval)		(0.4, 3.3)	(0.6, 2.7)	(0.2, 2.4)
	P-Value		**0.02**	**0.002**	**0.02**
Lactobacillus acidophilus	Probiotics and prebiotics	0.7±2.6	6.3±7.5***	7.2±7.6***	5.7±7.3**
	Placebo	1.5±3.6	2.1±5.1	1.1±3.1	1.7±4.0
	Mean difference		4.7	6.1	4.2
	(95% confidence interval)		(1.9, 7.5)	(3.6, 8.7)	(1.8, 6.5)
	P-Value		**0.001**	**<0.001**	**<0.001**
Lactobacillus	Probiotics and prebiotics	1.6±4.4	5.0±7.2*	10.0±7.4***	11.2±6.4***
	Placebo	2.0±4.8	3.7±5.5	6.3±5.9	7.5±5.6
	Mean difference		1.4	4.1	4.1

	(95% confidence interval)		(-1.2, 4.0)	(1.3, 7.0)	(1.6, 6.5)
	P-Value		0.29	**0.005**	**0.001**
Bifidobacterium longum	Probiotics and prebiotics	9.2±6.5	12.7±5.6**	14.0±4.4***	12.7±5.7***
	Placebo	10.5±6.2	10.3±5.8	11.1±5.1	11.6±5.2
	Mean difference		3.2	4.0	2.0
	(95% confidence interval)		(0.3, 6.0)	(1.3, 6.7)	(-0.5, 4.6)
	P-Value		**0.03**	**0.005**	0.12

Data are expressed as mean ± SD. Wilcoxon rank sum test or t-test was used to compare differences in beneficial intestinal bacteria between groups at baseline and within groups; covariance analysis was used to compare differences from baseline in beneficial intestinal bacteria between groups. * $P < 0.05$, ** $P < 0.01$, *** $P < 0.001$ for the difference from baseline within groups.

Table S5 Differences of SCFA between groups and within-group

SCFA		Baseline	D31	D61	D91
Total acids (μmol/g)	Probiotics and prebiotics	80.6±30.4	63.6±42.4**	61.4±33.4***	57.2±28.7***
	Placebo	83.4±30.2	89.4±49.6	83.9±35.5	87.3±49.1
	Mean difference		-20.3	-21.4	-27.5
	(95% confidence interval)		(-38.8, -2.0)	(-34.9, -7.9)	(-42.7, -12.3)
	P-Value		**0.03**	**0.002**	**0.001**
Acetate (%)	Probiotics and prebiotics	62.9±9.1	79.7±16.9***	77.6±15.1***	74.9±14.5***
	Placebo	63.1±8.4	63.4±9.8	64.8±7.9	63.7±9.1
	Mean difference		19.8	12.5	12.4
	(95% confidence interval)		(13.1, 26.5)	(6.6, 18.4)	(7.1, 17.6)
	P-Value		**<0.001**	**<0.001**	**<0.001**
Propionate (%)	Probiotics and prebiotics	16.6±6.1	9.9±9.6***	10.5±9.2***	12.1±9.1*
	Placebo	17.5±7.0	17.9±8.1	18.3±8.4	18.0±8.5
	Mean difference		-6.6	-7.3	-5.3
	(95% confidence interval)		(-9.9, -3.2)	(-10.7, -4.0)	(-8.6, -1.9)
	P-Value		**<0.001**	**<0.001**	**0.002**
Butyrate (%)	Probiotics and prebiotics	15.8±7.3	8.5±10.3***	10.2±10.5***	10.6±9.7**

	Placebo	15.4±6.8	14.9±7.6	13.9±6.4	14.8±6.7
	Mean difference		-5.6	-3.8	-3.9
	(95% confidence interval)		(-9.2, -2.1)	(-7.1, -0.5)	(-7.1, -0.6)
	P-Value		**0.002**	**0.03**	**0.02**
Isobutyrate (%)	Probiotics and prebiotics	1.3±1.2	0.6±0.6***	0.5±0.5***	0.6±0.9***
	Placebo	1.1±1.1	1.0±1.0	0.8±0.7	0.9±1.0
	Mean difference		-0.5	-0.3	-0.3
	(95% confidence interval)		(-0.8, -0.2)	(-0.6, -0.1)	(-0.6, 0.1)
	P-Value		**0.001**	**0.006**	0.12
Valerate (%)	Probiotics and prebiotics	1.5±1.5	0.6±0.9***	0.7±1.0***	0.7±1.4***
	Placebo	1.3±1.4	1.2±1.3	1.0±1.2	1.1±1.2
	Mean difference		-0.6	-0.4	-0.4
	(95% confidence interval)		(-1.0, -0.2)	(-0.8, 0.1)	(-0.8, 0.007)
	P-Value		**0.003**	0.09	0.05
Isovalerate (%)	Probiotics and prebiotics	2.0±1.9	0.8±0.8***	0.6±0.8***	1.0±1.6***
	Placebo	1.6±1.6	1.5±1.4	1.2±1.1	1.5±1.8
	Mean difference (95% confidence interval)		-0.6 (-1.1, -0.1)	-0.5 (-1.0, 0.04)	-0.4 (-1.1, 0.2)
	P-Value		**0.02**	0.07	0.20

Data are expressed as mean ± SD. Wilcoxon rank sum test or t-test was used to compare differences between groups at baseline and within groups; covariance analysis was used to compare differences in SCFAs from baseline between groups. * $P < 0.05$, ** $P < 0.01$, *** $P < 0.001$ for the difference from baseline within groups. SCFA, short-chain fatty acid.

16S rRNA gene sequencing data are available in the Figshare public repository (https://doi.org/10.6084/m9.figshare.25283461).
